# Supplementary material for: Declining Radial Growth in Major Western Carpathian Tree Species: Insights from Three Decades of Temperate Forest Monitoring
Source: Plants (Basel). 2023 Dec 6;12(24):4081. doi: 10.3390/plants12244081 (PMC10747720; doi:10.3390/plants12244081)
Supplement: Supplementary file 1 [file plants-12-04081-s001.zip › Figure_S1_S2.pdf]

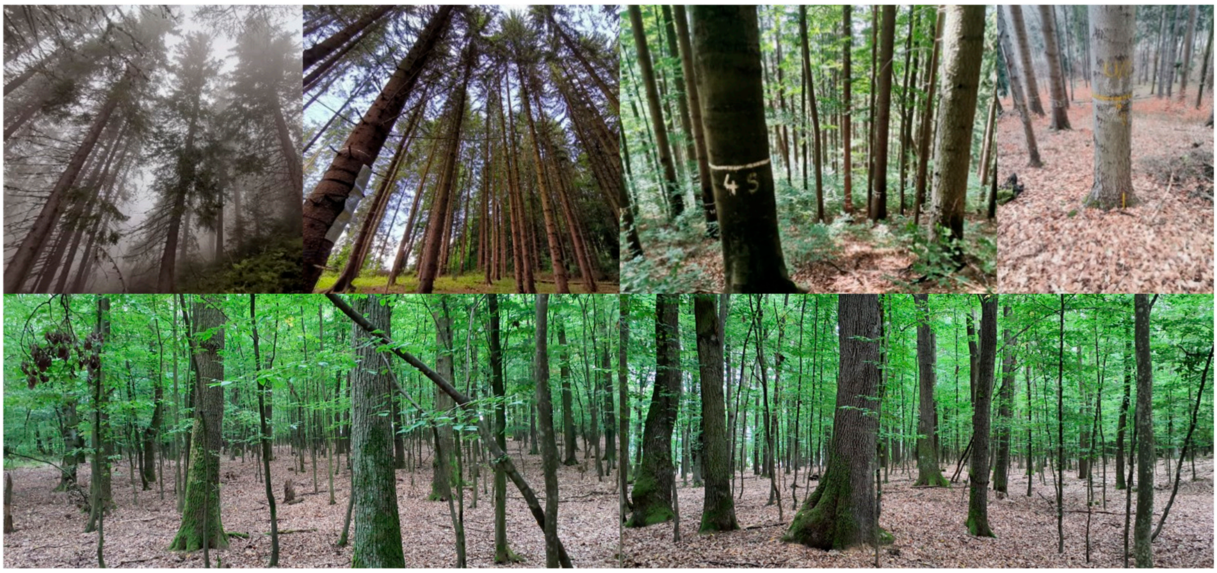

Figure S1 Forest structures typical for Western Carpathians examined in the study, from mountain spruce forests (upper left corner) trough mixed beech-fir dominated mountain forests (upper right corner) to sessile oak dominated foothills.

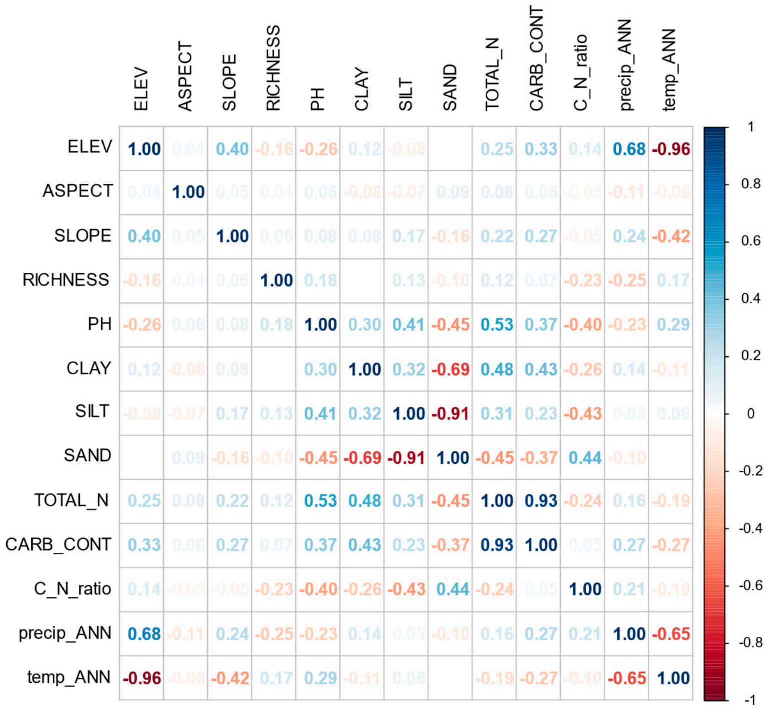

Figure S2 Correlation matrix of environmental variables. Values represent Pearson correlation coefficient.
