# Supplementary material for: Declining Radial Growth in Major Western Carpathian Tree Species: Insights from Three Decades of Temperate Forest Monitoring
Source: Plants (Basel). 2023 Dec 6;12(24):4081. doi: 10.3390/plants12244081 (PMC10747720; doi:10.3390/plants12244081)
Supplement: Supplementary file 1 [file plants-12-04081-s001.zip › Table_S1.pdf]

Table S1 Basic site description of permanent monitoring plots (ICP Forests Level I) analysed in the study

ICP-Forests Level I plot	t0	t_end	Elevation	Aspect	Slope	Precipit mean ann 1950-2022	Temp mean ann 1950-2022	Species and number of original trees	Tree Species richness	DBH mean_t0	DBH mean t_end	PH(H2O)	CLAY	SILT	SAND	TOTAL_N	Corg	C/N
[ID]	[yr]	[yr]	[m asl]	[deg]	[%]	[mm]	[°C]	[pcs]	[-]	[cm]	[cm]	[-]	[%]	[%]	[%]	[$g.kg^{-1}$]	[$g.kg^{-1}$]	[-]
A7	1989	2023	169	316	0.6	516	9.8	PS97	1	24.1±4	32±4.3	4.7	5.5	1.1	93.4	0.5	10.0	20.0
A8	1989	2023	187	308	0.6	518	9.7	PS65	1	22.2±3.5	28.1±3.9	5.4	5.7	2.6	91.7	0.3	8.3	27.7
B7	1989	2023	210	5	0.0	555	9.8	PS75	1	22.2±4.6	29.4±6.3	4.2	5.8	4.9	89.3	0.7	15.5	22.1
B8	1989	2023	304	5	8.6	562	9.1	FS56	2	20±3.8	36.2±7.4	7.1	30.8	54.8	14.4	6.3	85.7	13.6
C10	1989	2023	121	190	0.3	523	10.4	QP1	6	17.2±6.9	24.7±9.5	7.4	16.8	57.5	25.7	4.8	56.6	13.4
C7	1989	2023	289	304	23.3	595	9.2	FS78_QP2	2	21.7±8.4	35.5±8.4	7.7	31.5	67.2	1.2	8.8	117.9	11.8
D13	1989	2023	108	51	0.7	515	10.7	QP2	3	17±6.3	26.8±12.7	7.6	30.8	66.0	3.2	5.5	57.0	10.4
E5	1989	2023	322	267	21.6	734	8.7	FS3_QP34	5	18.5±6.4	29.7±10.3	6.8	26.4	64.0	9.6	3.5	46.6	13.3
E6	1989	2023	289	233	23.0	681	8.9	QP69	1	18.5±3.8	27.5±4.5	5.6	18.2	76.6	5.2	3.0	44.2	14.7
E7	1989	2023	536	130	13.4	688	8.3	FS35_PS2_QP1_PA13	8	25.9±8.2	38.5±10.9	6.8	23.9	73.9	2.2	9.6	114.1	11.9
F12a	2008	2023	111	340	0.5	521	10.7	QP31	4	18.8±4.1	23.7±4.9	4.8	6.7	11.0	82.3	3.1	36.1	11.6
F4	1989	2023	630	265	26.6	806	6.5	FS54	2	17.6±4.8	30.5±9	5.9	23.7	54.6	21.6	3.8	51.6	13.6
F5	1989	2023	431	316	13.1	769	8.2	FS24_PS23	8	23±7.3	40±11.5	5.7	31.6	64.0	4.4	2.9	35.3	12.2
F6	1989	2023	254	48	4.3	666	8.8	PS23_QP37	5	20.9±6.4	34±8.4	5.3	23.8	65.2	11.0	2.2	34.0	15.5
F7	1989	2023	264	264	4.4	604	9.3	QP26	3	15.1±4.3	26.3±7.8	4.5	15.2	76.2	8.6	2.2	28.8	13.1
G3	1989	2023	495	213	13.9	876	7.1	PS71	1	20.1±3.9	35.6±5.2	5.3	18.8	61.6	19.6	2.1	27.8	13.2
G4	1989	2023	325	180	11.6	805	8.3	PS2_QP2_PA56	4	24.5±6.1	42.1±11.7	4.7	17.5	76.1	6.4	2.1	35.6	17.0
G5	1989	2023	625	335	14.5	882	6.7	FS6_PS52	2	32.4±7	41.2±8.5	7.1	34.0	62.6	3.4	4.4	64.9	14.8
G6	1989	2023	450	109	14.8	758	7.6	FS57_QP5	2	31.6±11.1	49.8±7.6	4.7	13.2	50.2	36.6	1.4	23.9	17.1
G8	1989	2023	490	9	15.6	649	7.8	FS61	1	15±4.5	25.2±8.3	4.5	5.2	43.9	51.0	3.0	47.7	15.9
H1	1989	2023	915	209	15.7	1111	4.8	FS2_PA79	2	25.1±5.9	41.8±7.5	4.3	12.7	26.5	60.8	4.1	87.9	21.4
H2	1989	2007	585	16	14.3	1085	7.0	AA1_PA49	2	41.6±6.6	48.6±6	4.2	19.7	52.0	28.3	2.3	42.5	18.5
H2a	2008	2020	585	16	14.3	1085	7.0	PA50	1	39.1±4.6	43.3±5.5	4.2	19.7	52.0	28.3	2.3	42.5	18.5
H3	1989	2023	476	307	18.2	908	7.4	FS69	1	30.7±5.8	40.7±7.2	6.6	31.1	55.7	13.2	3.7	46.7	12.6
H4	1989	2023	499	132	20.8	914	7.2	FS1_PS34_PA37	3	24.7±8.1	30.3±8.6	6.8	26.6	70.7	2.7	4.5	85.4	19.0
H5	1989	2023	759	145	35.1	882	5.5	FS15_PA1	3	24±5.9	34.6±7.4	5.0	40.8	54.3	4.8	4.0	63.9	16.0
H7	1989	2023	932	335	10.3	858	5.5	FS59	2	28.4±13.6	38.6±17	4.3	28.4	49.5	22.1	8.3	100.3	12.1
H8	1989	2023	604	276	20.2	732	7.1	FS51_QP1	2	22.1±6.8	30.2±9.2	4.9	15.2	59.4	25.3	2.5	50.0	20.0
H9	1989	2023	248	273	27.3	664	9.6	FS43_QP1	5	15.8±4.5	27.6±9.4	5.8	7.3	46.9	45.7	3.2	48.6	15.2
I10	1989	2023	237	174	9.4	555	9.8	QP17	4	20.2±5.8	38.6±7.7	5.4	39.2	58.3	2.5	4.1	55.2	13.5
I2	1989	2020	461	21	24.9	1009	7.4	PA58	1	30.5±8.2	38.6±13.6	4.0	12.8	46.6	40.6	1.6	24.5	15.3
I4	1989	2023	804	168	29.4	971	4.7	FS8_AA4_PA51	4	29.1±6.1	41±9.2	4.4	16.8	40.2	43.0	3.0	49.6	16.5
I8	1989	2023	514	244	24.3	683	7.3	FS54_AP1	3	17.3±5.4	26.8±8.8	6.9	34.3	53.7	12.0	4.8	65.4	13.6
I9	1989	2023	575	350	5.8	688	7.2	FS6_QP40	3	16.5±5.5	26.1±9.3	4.8	13.5	66.6	19.9	2.2	28.7	13.0
J10	1989	2023	202	324	9.7	566	9.8	FS9_QP7	6	21.4±5.8	33.1±8.8	5.4	10.7	65.5	23.8	1.7	24.3	14.3

Notes
AA *Abies alba*
FS *Fagus sylvatica*
PA *Picea abies*
PS *Pinus sylvestris*
QP *Quercus petraea*

ICP-Forests Level I plot	t0	t_end	Elevation	Aspect	Slope	Precipit mean ann 1950-2022	Temp mean ann 1950-2022	Species and number of original trees	Tree Species richness	DBH mean_t0	DBH mean t_end	PH(H2O)	CLAY	SILT	SAND	TOTAL_N	Corg	C/N
[ID]	[yr]	[yr]	[m asl]	[deg]	[%]	[mm]	[°C]	[pcs]	[-]	[cm]	[cm]	[-]	[%]	[%]	[%]	[$g.kg^{-1}$]	[$g.kg^{-1}$]	[-]
J2	1989	2016	668	331	23.9	1135	6.3	PA53	1	29.3±7.6	38.5±9.5	4.2	24.2	51.5	24.3	2.2	32.1	14.6
J3	1989	2023	817	218	36.7	1082	5.3	FS29_PA45	3	25.1±6	35.4±6.7	6.6	32.0	57.0	11.1	5.7	75.9	13.3
J4	1989	2023	573	2	27.5	1052	7.3	FS29_AA2_PA42	5	23.1±8.9	36.7±12.4	6.1	37.6	60.1	2.3	3.2	41.4	12.9
J5	1989	2014	1012	208	26.5	1047	3.9	FS4_PA48	3	31.5±8.7	45.1±14	5.8	39.4	57.4	3.3	5.4	72.1	13.4
J6	1989	2023	720	21	28.7	1055	6.4	FS51_AA3_PA14	3	27.3±11.3	36.8±10.9	7.0	23.9	70.0	6.1	7.3	100.6	13.8
J7	1989	1996	586	66	21.7	793	7.3	FS50	1	35.3±11.4	36.8±12.1	5.8	9.9	68.7	21.3	2.0	26.8	13.4
J7a	1997	2016	586	66	21.7	793	7.3	FS50	1	29.4±4.9	34.6±4.2	5.8	9.9	68.7	21.3	2.0	26.8	13.4
J7b	2017	2023	635	113	5.9	781	6.9	FS50	1	16.9±3.7	18.8±4.5	5.8	9.9	68.7	21.3	2.0	26.8	13.4
J8	1989	2023	450	315	7.8	689	8.0	QP33_	2	16.8±3.2	25.8±6.5	5.2	14.2	48.5	37.3	2.1	32.3	15.4
K1	1989	2023	847	265	11.5	1158	5.1	PA79	1	29.4±7.9	42.5±9.7	3.6	28.3	46.0	25.7	7.5	155.2	20.7
K10	1989	2023	436	116	24.3	536	7.9	QP67	3	20.5±6.7	29.9±6.5	5.7	17.0	38.0	45.0	1.4	19.3	13.8
K2	1989	2023	862	331	8.1	971	4.3	PA71	1	28.6±6.6	38.8±7	4.0	44.0	54.8	1.2	2.6	40.6	15.6
K4	1989	2023	569	303	14.5	1004	7.1	PS4_PA83	2	21.7±5.7	39.1±5.1	5.2	34.2	63.2	2.6	4.5	72.4	16.1
K5	1989	2002	662	307	34.6	1056	6.5	PA57	1	28.7±5.6	31.8±7.3	7.1	21.9	75.0	3.1	9.9	142.2	14.4
K5a	2003	2023	662	307	34.6	1056	6.5	FS12_PA45	3	29.2±6.9	33.9±8	7.1	21.9	75.0	3.1	9.9	142.2	14.4
K6	1989	2001	555	161	15.8	849	6.6	PS31_PA20	3	31.5±10.5	35.3±9.6	6.9	18.3	65.5	16.2	7.4	111.3	15.0
K6a	2003	2023	555	161	15.8	849	6.6	PS48_PA5	3	25.6±7.5	33.8±7.1	6.9	18.3	65.5	16.2	7.4	111.3	15.0
L10	1989	2005	259	181	7.7	548	8.9	QP54	1	17.2±4	20.3±4.3	5.0	24.9	45.1	30.0	2.4	36.5	15.2
L2	1989	2023	1037	159	15.6	1021	4.1	AA1_PA77	2	29.9±6.1	35.7±6.3	4.1	15.7	51.7	32.6	2.2	33.7	15.3
L3	1989	2016	900	74	20.2	966	4.7	PS1_PA60	2	33.8±8	44.5±8.2	4.0	20.1	61.6	18.3	2.7	44.8	16.6
L5	1989	2023	1167	181	17.5	1022	2.6	FS3_PA71	3	23.7±6.6	39.6±8.2	4.4	7.7	31.2	61.1	3.5	40.3	11.5
L6	1989	2023	855	325	25.9	903	5.3	FS55_PA5	2	15.6±4	27.2±8.1	5.0	9.4	56.5	34.2	3.6	50.2	13.9
L7	1989	2023	1256	346	11.8	790	2.4	PA46	1	49.7±16.1	65.2±11.6	4.8	35.5	37.9	26.6	6.3	72.3	11.5
L8	1989	2023	505	166	20.9	688	7.5	FS54	1	29.2±10.4	41±13.5	4.6	9.6	47.4	43.1	1.3	23.5	18.1
L9	1989	2023	372	253	17.7	564	8.1	QP7_	3	16.9±5.2	24.3±7.7	5.3	16.8	72.1	11.1	2.0	27.5	13.8
M2	1989	2015	794	20	19.7	1073	5.2	PA53	1	30.5±7.1	42.4±10.6	4.5	19.8	31.6	48.7	3.1	58.5	18.9
M5	1989	2013	1310	32	28.3	1041	2.0	PA56	1	32±8.8	36.2±9.7	4.2	11.0	32.9	56.1	1.7	25.9	15.2
M7	1989	2023	627	21	21.9	820	7.0	FS22_AA30_PA12	3	36.8±11.5	51.2±14.1	4.0	20.3	39.1	40.6	3.6	57.5	16.0
M8	1989	2023	312	260	16.3	651	8.7	FS11_QP18	5	28.1±9.5	40.2±13	4.5	10.8	51.8	37.5	1.5	19.9	13.3
M9	1989	2023	230	145	15.4	550	9.3	QP28_AP41	4	22.6±7	35.8±8.9	5.0	32.8	60.1	7.2	2.9	31.7	10.9
N10	1989	2017	323	263	14.4	541	8.7	FS1_QP39	3	24.3±8	32.8±10.1	4.7	15.7	45.5	38.8	1.3	20.5	15.8
N10a	2022	2023	377	314	22.8	541	8.4	FS23_QP27	2	39.9±7.8	40.3±7.9	4.7	15.7	45.5	38.8	1.3	20.5	15.8
N3	1989	2013	1123	143	9.5	1456	2.7	PA58	1	46.2±12.2	47.4±12.7	4.0	10.1	36.2	53.8	2.1	29.7	14.1
N4	1989	2023	779	61	10.4	1302	6.1	PS2_PA55	2	32.3±5.8	41.2±7.5	5.1	12.0	58.2	29.8	1.0	17.8	17.8
N5	1989	2023	1009	337	22.1	1088	4.6	PA80	1	23.4±5.7	35.7±7.8	4.5	16.6	41.8	41.6	4.3	73.5	17.1
N6	1989	2023	928	100	12.1	878	4.6	FS13_AA12_PA71	3	28.5±8.7	37.5±10.6	5.0	28.1	69.9	2.1	4.7	77.2	16.4
N8	1989	2023	296	54	15.1	593	8.9	FS39_PS19_PA3	5	19.1±8.8	27.1±8.5	5.4	11.6	58.1	30.4	1.7	29.6	17.4
O4	1989	2023	790	141	1.3	1565	7.8	PS10_PA51	2	34.5±10.6	42±9.6	4.0	20.5	51.1	28.3	2.5	56.6	22.6
O5	1989	2023	1093	287	31.6	1033	3.5	PS1_PA75	4	30.7±7.2	36.9±8.7	6.1	15.5	78.9	5.6	6.8	104.8	15.4

O6	1989	2015	891	315	22.0	919	5.1	FS11_PA53	2	34.9±12.8	48.2±5.2	4.5	10.5	44.6	45.0	2.3	45.7	19.9
O6a	2022	2023	938	313	19.4	921	4.8	FS42_PA7	3	22±6	22.6±6.1	4.5	10.5	44.6	45.0	2.3	45.7	19.9
O7	1989	2016	587	332	26.7	595	6.8	FS48	5	26.8±10.6	39.1±13.5	5.9	9.9	51.9	38.2	2.0	24.6	12.3
ICP-Forests Level I plot	t0	t_end	Elevation	Aspect	Slope	Precipit mean ann 1950-2022	Temp mean ann 1950-2022	Species and number of original trees	Tree Species richness	DBH mean_t0	DBH mean t_end	PH(H2O)	CLAY	SILT	SAND	TOTAL_N	Corg	C/N
[ID]	[yr]	[yr]	[m asl]	[deg]	[%]	[mm]	[°C]	[pcs]	[-]	[cm]	[cm]	[-]	[%]	[%]	[%]	[g.kg^{-1}]	[g.kg^{-1}]	[-]
O7a	2017	2023	557	348	19.9	596	7.0	FS6_QP42	3	33±8.4	35±9	5.9	9.9	51.9	38.2	2.0	24.6	12.3
P2	1989	2023	611	144	23.9	790	6.5	AA2_PA44	6	21.3±8.3	38.8±18.8	5.0	20.5	73.2	6.3	4.2	50.2	12.0
P3	1989	2002	754	41	1.4	953	5.7	PS7_PA56	2	32.2±7.7	32.6±5.8	5.0	34.4	47.6	18.0	5.9	86.1	14.6
P5	1989	2023	654	307	17.2	742	6.0	FS2_PS3_AA13_PA39	5	21.6±5.6	27.5±8.6	7.2	20.0	77.3	2.7	10.8	151.1	14.0
P6	1989	2007	635	366	20.2	670	5.9	FS30__PA1	5	20.6±7.2	27.2±10.7	4.7	8.4	37.1	54.5	2.3	39.9	17.3
P6a	2011	2023	643	358	19.5	670	5.9	FS57_PA3	2	37.3±7.2	38.9±7.2	4.7	8.4	37.1	54.5	2.3	39.9	17.3
R2	1989	2023	709	350	15.8	851	6.5	PA65	1	20.8±4.6	30±8	4.5	32.5	62.2	5.3	5.1	70.7	13.9
R4	1989	2023	714	259	4.6	799	5.7	PS1_AA34_PA31	5	28±6.4	45.9±11.3	5.6	27.3	44.2	28.5	3.9	52.8	13.5
R5	1989	2023	507	21	18.7	725	7.6	PA72	1	23.7±5.7	35.2±6.1	4.3	14.3	56.1	29.5	2.2	38.5	17.5
R6	1989	2002	802	163	23.3	808	5.7	FS10_AA2_PA53	3	32.2±9.4	38.7±8.9	4.1	21.1	59.4	19.4	5.0	92.1	18.4
R6a	2003	2023	811	175	26.3	808	5.7	FS22_AA3_PA25	4	35.4±12.2	42.7±13.5	4.1	21.1	59.4	19.4	5.0	92.1	18.4
S2	1989	2023	479	149	12.8	826	7.7	FS35_PA3	3	22.9±6.4	31.9±10.2	5.0	15.1	49.2	35.8	4.7	63.5	13.5
S4	1989	2023	791	263	15.4	816	5.7	PA63	4	24.8±6.9	34.5±7.3	4.5	13.1	41.1	45.8	1.3	20.5	15.8
S5	1989	2023	493	203	5.8	751	7.6	PS27_PA42	2	29.4±6.1	39.5±8.4	6.2	22.6	53.6	23.8	4.3	72.5	16.9
S6	1989	2023	505	112	23.6	767	7.6	FS46_PS1_PA5	4	25.2±5.9	36±8.2	4.2	32.6	46.9	20.5	6.7	106.1	15.8
S7	1989	2023	737	3	20.9	587	5.4	FS34_AA31_PA1	3	33.9±7.2	43.2±7.7	5.8	33.0	61.8	5.2	2.8	40.6	14.5
T3	1989	1997	732	356	18.9	806	5.9	AA25	1	33.2±9.6	36.7±11.4	5.0	13.6	50.9	35.5	2.9	43.3	14.9
T3_2	2002	2023	732	356	18.9	806	5.9	FS11_AA54_PA3	5	37.8±14	37.2±13.2	5.0	13.6	50.9	35.5	2.9	43.3	14.9
T4	1989	2023	580	106	16.1	765	7.0	AA65	6	31.4±9.7	45.7±12.2	4.8	11.3	42.3	46.4	2.3	32.5	14.1
T5	1989	2023	750	232	30.7	738	5.8	FS32_QP8_C	5	31.9±9.3	42.4±11	5.3	11.7	47.2	41.1	4.0	52.3	13.1
T6	1989	2023	884	330	30.4	756	5.1	FS40_AA4	4	41.3±10.8	49.4±11.9	4.4	14.2	55.1	30.7	4.0	59.4	14.9
U2	1989	2023	455	324	11.3	764	7.0	FS14_PS1_QP1_AA33_PA6	7	27.7±8.6	45.2±14	5.0	13.3	41.4	45.3	2.9	41.1	14.2
U3	1989	2023	512	124	14.6	792	7.0	FS28_AA43_PA4	6	32.4±8.5	41.8±11.9	4.6	14.4	50.3	35.3	2.5	38.4	15.4
U4	1989	2023	371	14	6.9	812	9.1	QP15	4	25.7±5.9	34.9±9.3	5.5	11.2	41.4	47.5	2.6	32.7	12.6
U5	1989	2023	367	250	11.3	664	7.9	FS5_QP2	4	34.4±11.6	39.8±9.9	5.3	12.0	51.8	36.1	1.9	23.9	12.6
U6	1989	2023	383	322	10.6	711	8.7	PS64	1	32.2±5.4	35.7±6.8	7.6	25.3	70.6	4.0	6.8	25.0	3.7
U7	1989	2023	217	188	0.1	594	9.2	QP55	1	40.6±6.8	52±8.7	6.4	27.4	66.2	6.5	4.9	72.4	14.8
V2	1989	2023	391	5	13.8	753	7.6	FS50	6	31.4±13.3	53.7±17.7	4.5	20.1	72.2	7.7	2.9	31.8	11.0
V3	1989	2023	288	32	12.6	699	8.1	FS47	3	17.9±5.3	33.4±8.6	4.6	10.9	79.1	10.0	1.6	24.9	15.6
V4	1989	2023	331	48	12.3	710	8.2	FS54_QP4	2	34.5±7.7	44.8±10.1	5.4	13.5	70.1	16.4	3.0	30.3	10.1
V5	1989	2019	749	75	12.2	819	6.2	FS67	2	37.1±6.6	42.7±3.4	4.7	30.3	49.4	20.3	5.2	66.4	12.8
V7	1989	2023	417	225	10.6	678	8.3	FS68_QP1	2	32.6±6.7	42.7±10.8	5.0	11.7	80.4	7.8	2.4	33.7	14.0
W6	1999	2023	634	246	10.8	875	6.7	FS64	2	36.9±9	45.8±10.2	5.2	16.1	74.5	9.4	1.5	30.4	20.3
X3	1989	2023	341	59	24.4	812	7.8	FS48	2	20.5±5.3	34.2±12.3	5.1	27.6	64.1	8.3	1.7	19.4	11.4
X4	1989	2015	385	320	12.0	916	6.7	FS58	1	43.8±10	54.5±9.3	4.8	12.2	58.3	29.5	1.2	15.1	12.6
Y3	1989	2022	551	273	12.1	916	6.7	FS52	2	14.3±3.8	28.7±9.5	5.1	20.7	71.2	8.1	2.6	33.9	13.0
Y4	1989	2015	295	303	12.2	829	8.1	FS50	2	35.5±6.6	48.4±6.8	4.8	17.4	78.0	4.6	1.2	13.7	11.4

Y4a	2023	2023	325	261	10.8	835	7.9	FS34	2	24.8±6.5	24.8±6.5	4.8	17.4	78.0	4.6	1.2	13.7	11.4
Z2	1989	2023	387	17	13.4	781	7.3	FS54	1	29.7±12.6	39.1±17.6	5.1	28.5	66.5	5.0	1.5	18.7	12.5
Z3	1989	2023	250	305	7.3	741	8.4	QP7	4	33.9±8.7	45.7±11.7	4.4	12.3	40.4	47.3	2.1	27.9	13.3
Z4	1989	2020	221	311	18.7	705	8.6	FS50	1	32.6±8.3	42.4±7.3	4.4	12.0	40.7	47.3	1.8	31.3	17.4
Z4a	2023	2023	233	294	22.7	705	8.5	FS35_PS1_QP14	3	25.7±6.8	25.9±6.8	4.4	12.0	40.7	47.3	1.8	31.3	17.4
